# Supplementary material for: Skin T cells maintain their diversity and functionality in the elderly
Source: Commun Biol. 2021 Jan 4;4:13. doi: 10.1038/s42003-020-01551-7 (PMC7782613; doi:10.1038/s42003-020-01551-7)
Supplement: Supplementary file 2 — Supplementary Information [file 42003_2020_1551_MOESM2_ESM.pdf]

Supplementary information

Title: Skin T cells maintain their diversity and functionality in the elderly

Authors:

Hanako Koguchi-Yoshioka[1,2], Elena Hoffer[3], Stanley Cheuk[3], Yutaka Matsumura[1], Sa Vo[1], Petra Kjellman[4], Lucian Grema[4], Yosuke Ishitsuka[1], Yoshiyuki Nakamura[1], Naoko Okiyama[1], Yasuhiro Fujisawa[1], Manabu Fujimoto[1,2], Liv Eidsmo[3,4†], Rachael A. Clark[5†], and Rei Watanabe[1,2†].

†These authors jointly supervised this work.

Affiliations:

[1]Department of Dermatology, Faculty of Medicine, University of Tsukuba, Ibaraki, Japan

1-1-1 Tennodai, Tsukuba, Ibaraki 305-8575, Japan

[2]Department of Dermatology, Course of Integrated Medicine, Graduate School of Medicine, Osaka University, Osaka, Japan

2-2 Yamadaoka, Suita, Osaka, 565-0871

[3]Division of Rheumatology, Department of Medicine Solna, Karolinska Institutet, Solna, Sweden

Visionsgatan 18, L8, 171 76, Solna, Sweden

[4]Diagnostiskt Centrum Hud, Stockholm, Sweden

Apelbergsgatan 60, 111 37, Stockholm, Sweden

[5]Department of Dermatology, Brigham and Women's Hospital, Boston, MA, USA

75 Francis St, Boston, MA 02115, USA

Corresponding author: Rei Watanabe, MD, PhD

Department of Dermatology, Faculty of Medicine, University of Tsukuba, 1-1-1 Tennodai, Tsukuba, Ibaraki 305-8575, Japan

E-mail address: rwatanabe@md.tsukuba.ac.jp

Present address: Department of Dermatology, Course of Integrated Medicine, Graduate School of Medicine, Osaka University, Osaka, Japan

2-2 Yamadaoka, Suita, Osaka, 565-0871

Supplementary Figures

Supplementary Figure 1. Phenotypical and functional alteration of T cells in aged blood and skin

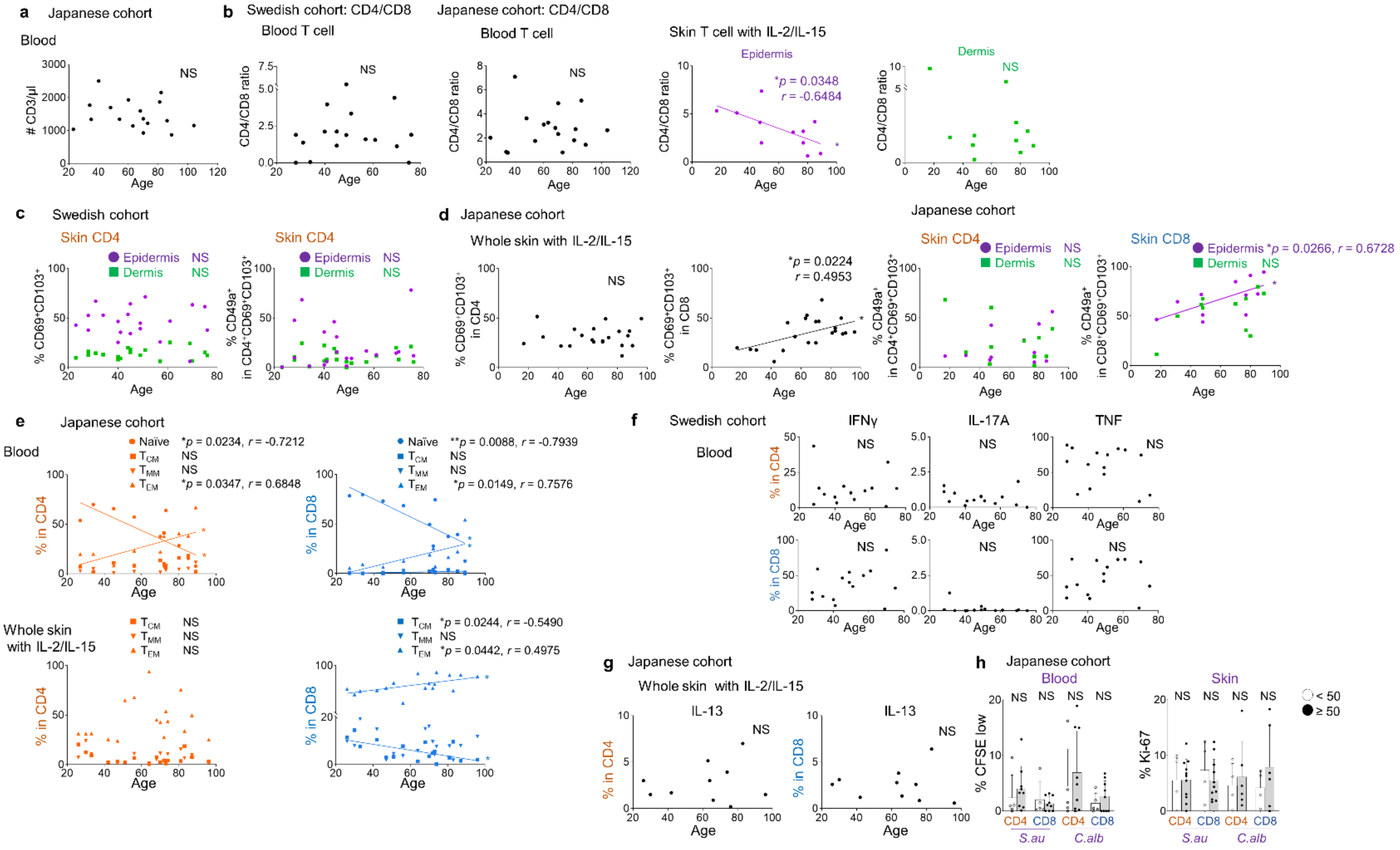

a) CD3⁺ numbers/μl blood (Japanese). N = 17.

b) CD4/CD8 ratio in blood, epidermal, and dermal T cells (Swedish and Japanese). Swedish: blood: n = 17; Japanese: blood: n = 17, skin: n = 11.

c) CD69/CD103 expression in CD4⁺ and CD49a expression in CD69⁺CD103⁺CD4⁺ from epidermis and dermis (Swedish). N = 20.

d) Whole skin with IL-2/IL-15: CD69/CD103 expression in CD4⁺ and CD8⁺ from whole skin (Japanese). N = 21. Skin CD4 and CD8: CD49a expression in CD69⁺CD103⁺CD4⁺ and CD69⁺CD103⁺CD8⁺ from epidermis and dermis (Japanese). N = 11.

e) Blood: The ratios of naïve T cells (CD45RA⁺CD45RO⁻), T_{CM} (CD45RO⁺CCR7⁺CD62L⁺), migratory memory T cells (T_{MM}, CD45RO⁺CCR7⁺CD62L⁻), and T_{EM} (CD45RO⁺CCR7⁻) in blood CD4⁺ and CD8⁺ (Japanese). N=10. Whole skin with IL-2/IL-15: T_{CM} (CCR7⁺CD62L⁺),

T_{MM} ($CCR7^+CD62L^-$), T_{EM} ($CCR7^-$ $CD62L^-$) in whole skin $CD4^+$ and $CD8^+$ (Japanese). N = 17.

f) The production ratios of IFNγ, IL-17A, and TNF in blood $CD4^+$ and $CD8^+$ (Swedish). N = 15.

g) IL-13 production from skin $CD4^+$ and $CD8^+$ (Japanese). N = 10.

h) Proliferative activities of T cells from blood and skin cultured with heat-killed *S.au* or *C.alb*, as in Fig. 2a. Blood T cells were evaluated by CFSE levels, and skin T cells, by Ki-67 expression. Error bars indicate standard deviations. Blood: n = 14; skin: *S.au*: n = 15, *C.alb*: n = 10.

Spearman rank correlation coefficients (two-tailed) were performed in a - g and Mann-Whitney test (two-tailed) was applied in h. NS: not significant, $^*p < 0.05$, $^{**}p < 0.01$.

Supplementary Figure 2. Subanalyses of T-cell phenotypes from the head vs the trunk and from the flap edge vs the transplant skin specimens of participants aged older than 50 years

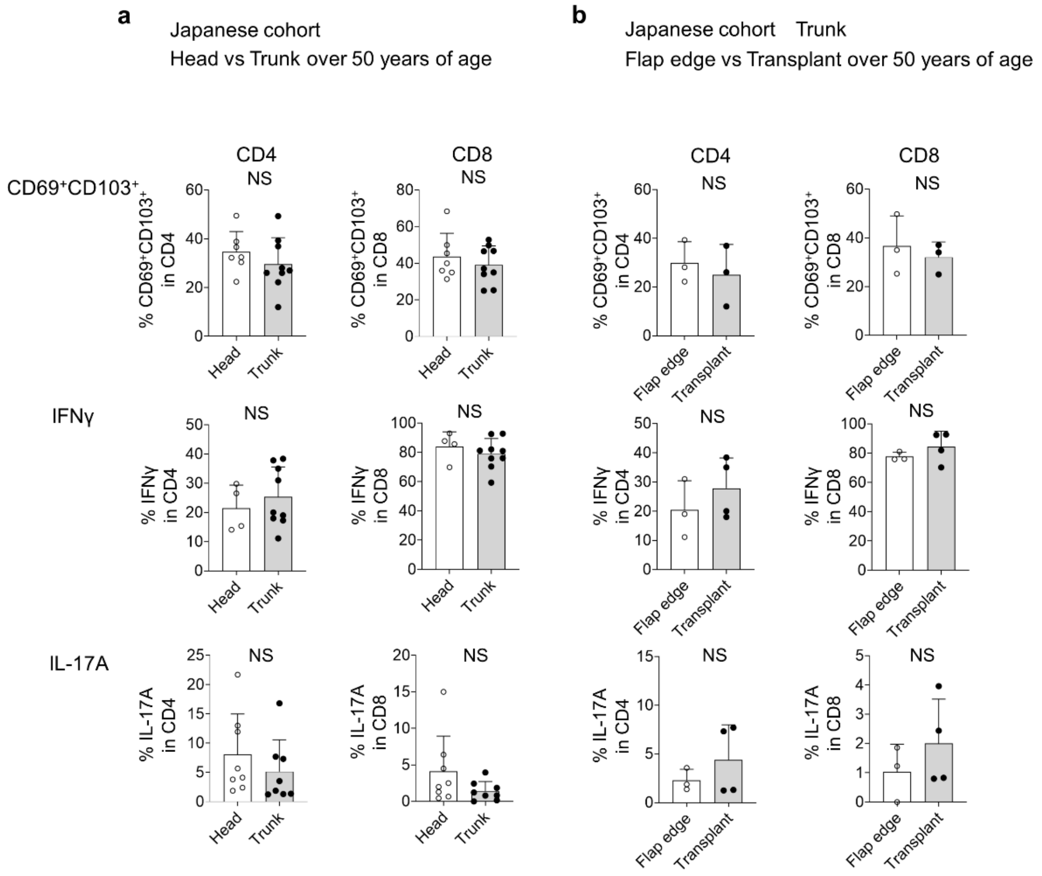

The samples are from Japanese participants. Error bars indicate standard deviations.

a) T_{RM} phenotypes and production of IFNγ and IL-17A were compared between T cells from the head (white dots) and the trunk (black dots) specimens. The participants were extracted from the analyses shown in Fig. 1d and Supplementary Figure 1d. $CD69^{+}103^{+}$: n = 16, IFNγ: n = 13, IL-17A: n = 16.

b) T_{RM} phenotypes and cytokine production profiles were compared between T cells from flap edge (white dots) and transplant samples (black dots). The participants were extracted from the analyses shown in Fig. 1d and Supplementary Figure 1d. $CD69^{+}103^{+}$: n = 6, IFNγ: n = 7, IL-17A: n = 7. NS: not significant.

Mann-Whitney tests (two-tailed) were performed.

Supplementary Figure 3. Gating strategies in flow cytometry

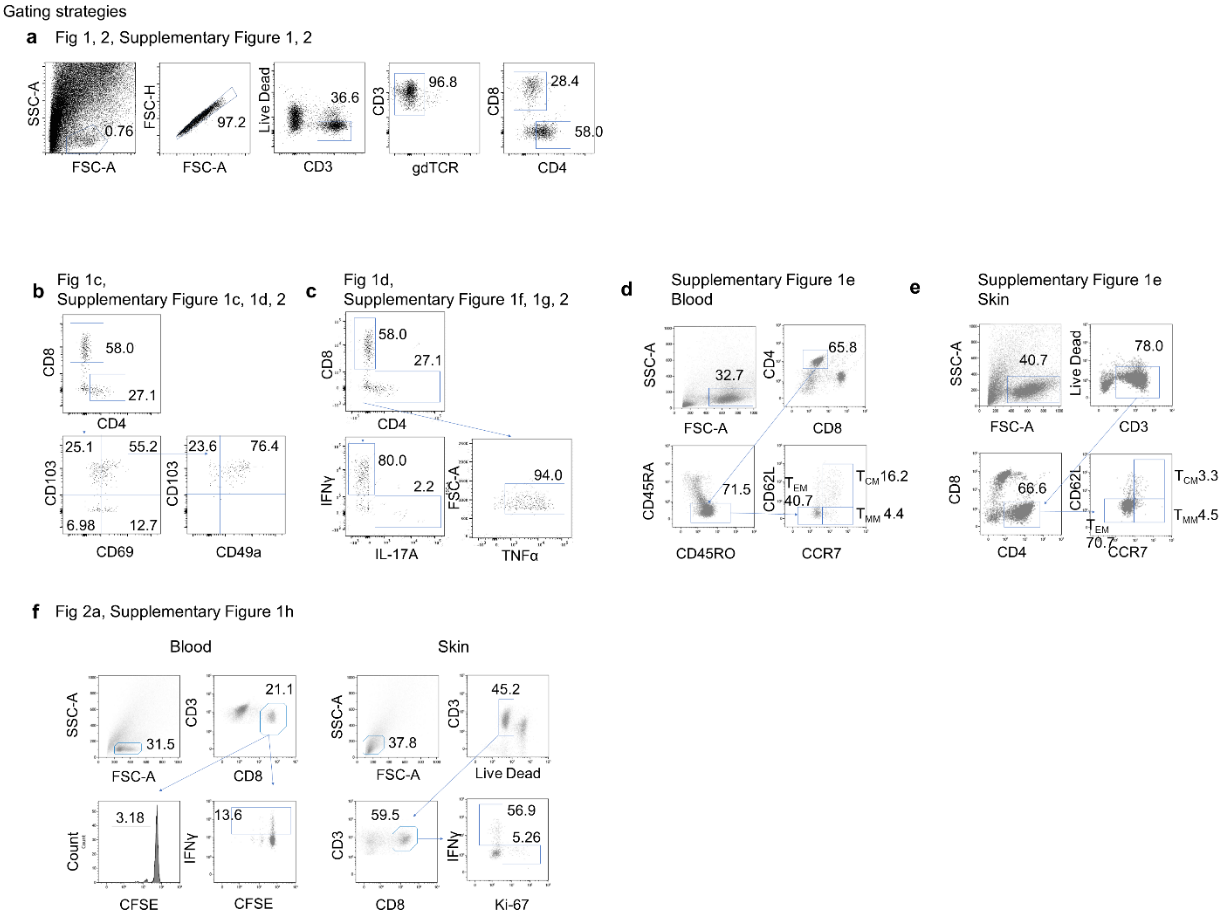

a) Identification of T cells, CD4$^+$, and CD8$^+$ cells in Fig. 1, 2, Supplementary Figure 1, and Supplementary Figure 2.
b) Identification of skin T_{RM} in Fig. 1c, Supplementary Figure 1c, 1d, and 2.
c) Cytokine production from T cells in blood, epidermis, and dermis in Fig. 1d, Supplementary Figure 1f, 1g, and 2.
d) Identification of blood T_{CM}, T_{MM}, and T_{EM} in Supplementary Figure 1e.
e) Identification of skin T_{CM}, T_{MM}, and T_{EM} in Supplementary Figure 1e.
f) CFSE reduction and cytokine production in blood T cells and Ki-67 expression and cytokine production in skin T cells in Fig. 2a and Supplementary Figure 1h.

Supplementary Table 1.

Lists of the participants. The participants whose data were used in the different analyses are shown in gray from the second time.

Body sites: head (H), trunk (T), upper extremities (U), and lower extremities (L).

Skin diseases: benign or in situ malignant (basal cell carcinoma [BCC] and squamous cell carcinoma in situ (actinic keratosis [AK]/Bowen.

Figure 1a (skin: n = 9 from Japanese)

Age	Body site	Skin disease	Flap edge or transplant
57	T	Benign	Edge
20	H	Benign	Edge
72	T	Benign	Edge
68	T	Benign	Edge
40	U	Benign	Edge
80	H	In situ malignant (BCC)	Edge
85	H	In situ malignant (BCC)	Edge
36	U	Benign	Edge
92	H	In situ malignant (BCC)	Edge

Figure 1b, 1c (skin: n = 20 from Swedish)

Age
49
49
70
69
41
45
40
57
51
44
40
45
76
75

23
31
61
28
34
28

Figure 1d IFNγ, IL-17A (skin: n = 18 from Swedish)

Age
49
49
70
69
41
45
40
57
51
44
40
75
23
31
61
28
34
28

Figure 1d TNF (skin: n = 16 from Swedish)

Age
49
49
70
69

41
45
40
57
51
75
23
31
61
28
34
28

Figure 1d IFNγ (skin: n = 18 from Japanese)

Age	Body site	Skin disease	Flap edge or transplant
72	T	Benign	Transplant
74	H	In situ malignant (BCC)	Transplant
64	T	Benign	Edge
96	H	In situ malignant (AK/Bowen)	Edge
30	U	Benign	Edge
87	T	Benign	Edge
86	H	In situ malignant (AK/Bowen)	Edge
83	H	In situ malignant (AK/Bowen)	Edge
42	T	In situ malignant (AK/Bowen)	Transplant
26	T	Benign	Edge
17	H	Benign	Edge
48	H	Benign	Edge
63	T	In situ malignant (AK/Bowen)	Edge
65	T	In situ malignant (AK/Bowen)	Edge
90	T	In situ malignant (AK/Bowen)	Edge
88	T	Benign	Transplant
70	T	Benign	Transplant
83	T	Benign	Transplant

Figure 1d IL-17A (skin: n = 21 from Japanese)

Age	Body site	Skin disease	Flap edge or transplant
72	T	Benign	Transplant
87	H	Benign	Edge
74	H	In situ malignant (BCC)	Transplant
64	T	Benign	Edge
96	H	In situ malignant (AK/Bowen)	Edge
30	U	Benign	Edge
87	T	Benign	Edge
86	H	In situ malignant (AK/Bowen)	Edge
83	H	In situ malignant (AK/Bowen)	Edge
42	T	In situ malignant (AK/Bowen)	Transplant
51	H	Benign	Edge
26	T	Benign	Edge
17	H	Benign	Edge
85	H	In situ malignant (AK/Bowen)	Edge
48	H	Benign	Edge
63	T	In situ malignant (AK/Bowen)	Edge
65	T	In situ malignant (AK/Bowen)	Edge
90	T	In situ malignant (AK/Bowen)	Edge
88	T	Benign	Transplant
70	T	Benign	Transplant
83	T	Benign	Transplant

Figure 1d TNF (skin: n = 16 from Japanese)

Age	Body site	Skin disease	Flap edge or transplant
72	T	Benign	Transplant
84	H	In situ malignant (BCC)	Transplant
74	H	In situ malignant (BCC)	Transplant
64	T	Benign	Edge
96	H	In situ malignant (AK/Bowen)	Edge
30	U	Benign	Edge
87	H	Benign	Edge
86	H	In situ malignant (AK/Bowen)	Edge

83	H	In situ malignant (AK/Bowen)	Edge
42	T	In situ malignant (AK/Bowen)	Transplant
51	H	Benign	Edge
26	T	Benign	Edge
17	H	Benign	Edge
85	H	In situ malignant (AK/Bowen)	Edge
48	H	Benign	Edge
87	T	Benign	Edge

Figure 2a, Supplementary Figure 1h (blood: n = 14 from Japanese)

Age
59
68
29
48
31
34
40
60
63
81
68
68
82
104

Figure 2A, Supplementary Figure 1H (*S.au*-injected skin: n = 15 from Japanese)

Age	Body site	Skin disease	Flap edge or transplant
25	T	Benign	Edge
20	H	Benign	Edge
34	H	Benign	Edge
60	T	In situ malignant (BCC)	Transplant
75	L	In situ malignant (AK/Bowen)	Edge
75	L	In situ malignant (AK/Bowen)	Edge

81	T	In situ malignant (AK/Bowen)	Transplant
96	L	In situ malignant (AK/Bowen)	Transplant
83	T	In situ malignant (BCC)	Transplant
93	T	In situ malignant (AK/Bowen)	Transplant
71	T	In situ malignant (BCC)	Transplant
91	L	In situ malignant (BCC)	Transplant
83	T	In situmalignant (BCC)	Transplant
84	T	In situ malignant (AK/Bowen)	Transplant
41	T	In situ malignant (BCC)	Transplant

Figure 2a, Supplementary Figure 1h (*C.alb*-injected skin: n = 10 from Japanese)

Age	Body site	Skin disease	Flap edge or transplant
93	T	In situ malignant (AK/Bowen)	Transplant
71	T	In situ malignant (BCC)	Transplant
83	T	In situ malignant (BCC)	Transplant
84	T	In situ malignant (AK/Bowen)	Transplant
41	T	In situ malignant (BCC)	Transplant
88	T	In situ malignant (AK/Bowen)	Transplant
76	T	In situ malignant (BCC)	Transplant
44	T	Benign	Edge
35	H	Benign	Edge
48	T	Benign	Edge

Figure 3, 4 (blood and skin: n = 16 from Japanese)

Age	Body site	Skin disease	Flap edge or transplant
46	T	Benign	Edge
57	T	Benign	Edge
73	T	In situ malignant (AK/Bowen)	Edge
57	U	Benign	Edge
66	H	In situ malignant (BCC)	Edge
41	T	Benign	Edge
79	H	Benign	Edge
20	H	Benign	Edge
48	T	Benign	Edge

90	H	In situ malignant (AK/Bowen)	Edge
83	T	Benign	Edge
90	H	In situ malignant (AK/Bowen)	Edge
88	H	In situ malignant (AK/Bowen)	Edge
42	T	Benign	Edge
84	T	In situ malignant (BCC)	Edge
26	T	Benign	Edge

Supplementary Figure 1a, 1b (blood: n = 17 from Japanese)

Age
23
34
40
60
63
68
81
86
89
82
104
70
48
70
35
54
73

Supplementary Figure 1b, 1c (blood (n = 17) or skin (n = 20) from Swedish)

Skin	Blood
Age	Age
49	49
49	49
70	70

69	69
41	41
45	45
40	40
57	57
51	51
44	
40	
45	45
76	76
75	75
23	
31	31
61	61
28	28
34	34
28	28

Supplementary Figure 1b, 1d (skin: n = 11 from Japanese)

Age	Body site	Skin disease	Flap edge or transplant
47	T	Benign	Edge
77	T	In situ malignant (BCC)	Transplant
31	T	Benign	Edge
70	L	In situ malignant (BCC)	Transplant
17	H	Benign	Edge
48	U	Benign	Edge
85	H	In situ malignant (AK/Bowen)	Edge
77	U	Benign	Edge
80	H	In situ malignant (BCC)	Edge
48	U	Benign	Edge
89	T	In situ malignant (AK/Bowen)	Transplant

Supplementary Figure 1d (whole skin: n = 21 from Japanese)

Age	Body site	Skin disease	Flap edge or transplant
72	T	Benign	Transplant
56	H	Benign	Edge
87	H	Benign	Edge
74	H	In situ malignant (BCC)	Transplant
64	T	Benign	Edge
96	H	In situ malignant (AK/Bowen)	Edge
83	H	In situ malignant (AK/Bowen)	Edge
81	H	In situ malignant (BCC)	Edge
51	H	Benign	Edge
70	T	In situ malignant (BCC)	Edge
63	T	In situ malignant (AK/Bowen)	Edge
65	T	In situ malignant (AK/Bowen)	Edge
90	T	In situ malignant (AK/Bowen)	Edge
88	T	Benign	Transplant
70	T	Benign	Transplant
83	T	Benign	Transplant
30	U	Benign	Edge
42	T	In situ malignant (AK/Bowen)	Transplant
26	T	Benign	Edge
17	H	Benign	Edge
48	H	Benign	Edge

Supplementary Figure 1e (blood: n = 10 from Japanese)

Age
27
34
45
56
70
72
73
80

85
89

Supplementary Figure 1e (skin: n = 17 from Japanese)

Age	Body site	Skin disease	Flap edge or transplant
72	T	Benign	Transplant
56	H	Benign	Edge
87	H	Benign	Edge
74	H	In situ malignant (BCC)	Transplant
64	T	Benign	Edge
96	H	In situ malignant (AK/Bowen)	Edge
30	U	Benign	Edge
83	H	In situ malignant (AK/Bowen)	Edge
68	L	In situ malignant (AK/Bowen)	Edge
42	T	In situ malignant (AK/Bowen)	Transplant
81	H	In situ malignant (BCC)	Edge
47	H	Benign	Edge
51	H	Benign	Edge
26	T	Benign	Edge
33	U	Benign	Edge
73	L	In situ malignant (BCC)	Edge
70	T	In situ malignant (BCC)	Edge

Supplementary Figure 1f (blood: n = 15 from Swedish)

Blood
Age
49
49
70
69
41
45
40
57

51
75
31
61
28
34
28

Supplementary Figure 1g IL-13 (skin: n = 10 from Japanese)

Age	Body site	Skin disease	Flap edge or transplant
74	H	In situ malignant (BCC)	Transplant
64	T	Benign	Edge
96	H	In situ malignant (AK/Bowen)	Edge
30	U	Benign	Edge
83	H	In situ malignant (AK/Bowen)	Edge
42	T	In situ malignant (AK/Bowen)	Transplant
26	T	Benign	Edge
76	T	In situ malignant (BCC)	Transplant
63	L	In situ malignant (AK/Bowen)	Edge
66	T	In situ malignant (BCC)	Transplant

Supplementary Figure 2a $CD69^{+}CD103^{+}$ (skin: n = 16 from Japanese)

Age	Body site	Skin disease	Flap edge or transplant
72	T	Benign	Transplant
56	H	Benign	Edge
87	H	Benign	Edge
74	H	In situ malignant (BCC)	Transplant
64	T	Benign	Edge
96	H	In situ malignant (AK/Bowen)	Edge
83	H	In situ malignant (AK/Bowen)	Edge
81	H	In situ malignant (BCC)	Edge
51	H	Benign	Edge
70	T	In situ malignant (BCC)	Edge
63	T	In situ malignant (AK/Bowen)	Edge

65	T	In situ malignant (AK/Bowen)	Edge
90	T	In situ malignant (AK/Bowen)	Edge
88	T	Benign	Transplant
70	T	Benign	Transplant
83	T	Benign	Transplant

Supplementary Figure 2a IFNγ (skin: n = 13 from Japanese)

Age	Body site	Skin disease	Flap edge or transplant
72	T	Benign	Transplant
74	H	In situ malignant (BCC)	Transplant
64	T	Benign	Edge
96	H	In situ malignant (AK/Bowen)	Edge
87	T	Benign	Edge
86	H	In situ malignant (AK/Bowen)	Edge
83	H	In situ malignant (AK/Bowen)	Edge
63	T	In situ malignant (AK/Bowen)	Edge
65	T	In situ malignant (AK/Bowen)	Edge
90	T	In situ malignant (AK/Bowen)	Edge
88	T	Benign	Transplant
70	T	Benign	Transplant
83	T	Benign	Transplant

Supplementary Figure 2a IL-17A (skin: n = 16 from Japanese)

Age	Body site	Skin disease	Flap edge or transplant
72	T	Benign	Transplant
87	H	Benign	Edge
74	H	In situ malignant (BCC)	Transplant
64	T	Benign	Edge
96	H	In situ malignant (AK/Bowen)	Edge
87	H	Benign	Edge
86	H	In situ malignant (AK/Bowen)	Edge
83	H	In situ malignant (AK/Bowen)	Edge
51	H	Benign	Edge
85	H	In situ malignant (AK/Bowen)	Edge

63	T	In situ malignant (AK/Bowen)	Edge
65	T	In situ malignant (AK/Bowen)	Edge
90	T	In situ malignant (AK/Bowen)	Edge
88	T	Benign	Transplant
70	T	Benign	Transplant
83	T	Benign	Transplant

Supplementary Figure 2b (skin: n = 7 from Japanese)

Age	Body site	Skin disease	Flap edge or transplant
63	T	In situ malignant (AK/Bowen)	Edge
65	T	In situ malignant (AK/Bowen)	Edge
90	T	In situ malignant (AK/Bowen)	Edge
72	T	Benign	Transplant
88	T	Benign	Transplant
70	T	Benign	Transplant
83	T	Benign	Transplant

Supplementary Table 2.

List of antibodies used for flow cytometry analyses.

Antibodies	Clone	Type	Species	Supplier
CD3	SK7	Monoclonal	Mouse	eBioscience
CD4	RPA-T4	Monoclonal	Mouse	eBioscience
CD8a	RPA-T8	Monoclonal	Mouse	eBioscience
CD62L	DREG56	Monoclonal	Mouse	eBioscience
CCR7	G043H7	Monoclonal	Mouse	BioLegend
CD45RA	HI100	Monoclonal	Mouse	BioLegend
CD45RO	UCHL1	Monoclonal	Mouse	BioLegend
CD69	FN50	Monoclonal	Mouse	BioLegend
CD49a	SR84	Monoclonal	Mouse	BD Biosciences
CD103	Ber-ACT8	Monoclonal	Mouse	BioLegend
IFNγ	4S.B3	Monoclonal	Mouse	BioLegend
IL-17A	BL168	Monoclonal	Mouse	BioLegend
TNFα	MAb11	Monoclonal	Mouse	BioLegend
Ki-67	Ki-67	Monoclonal	Mouse	BioLegend
IL-13	JES10-5A2	Monoclonal	Mouse	BioLegend

Supplementary Table 3.

List of the statistics and exact *p* and *r* values, mean ± standard deviations when applicable.

Figure 1a

Statistics: Spearman rank correlation coefficient

	p value (two-tailed)	*r* value
CD3 in epidermis	0.0028	0.8806
CD3 in dermis	0.9405	0.03405
CD3 in whole skin	0.2140	0.4603
CD4 in epidermis	0.0016	0.9093
CD4 in dermis	0.7961	-0.1022
CD4 in whole skin	0.1968	0.483
CD8 in epidermis	0.0206	0.7694
CD8 in dermis	0.6077	0.1975
CD8 in whole skin	0.2108	0.4642

Figure 1b

Statistics: Spearman rank correlation coefficient

	p value (two-tailed)	*r* value
CD4/CD8 in epidermis	0.0349	-0.4736
CD4/CD8 in dermis	0.1831	-0.3102

Figure 1c

Statistics: Spearman rank correlation coefficient

	p value (two-tailed)	*r* value
<%CD69$^+$CD103$^+$ in CD8>		
Epidermis	0.9523	0.01431
Dermis	0.0335	0.4768
<%CD49a$^+$ in CD69$^+$CD103$^+$CD8>		
Epidermis	0.0224	0.5075
Dermis	0.7476	-0.07681

Figure 1d

Statistics: Spearman rank correlation coefficient

	p value (two-tailed)	*r* value
<Epidermis>		
%IFNγ in CD4	0.1960	0.3405
%IL-17A in CD4	0.3795	-0.2343
%TNF in CD4	0.2094	-0.3568
%IFNγ in CD8	0.4402	0.2063
%IL-17A in CD8	0.0490	-0.4700
%TNF in CD8	0.4368	-0.2247
<Dermis>		
%IFNγ in CD4	0.8111	0.06485
%IL-17A in CD4	0.0284	-0.5527
%TNF in CD4	0.1630	-0.3943
%IFNγ in CD8	0.9366	0.02211
%IL-17A in CD8	0.0062	-0.6837
%TNF in CD8	0.9896	-0.00441
<Whole skin with IL-2/IL-15>		
%IFNγ in CD4	0.2589	-0.2808
%IL-17A in CD4	0.0042	-0.5973
%TNF in CD4	0.1420	0.3841
%IFNγ in CD8	0.0118	0.5483
%IL-17A in CD8	0.0196	-0.5049
%TNF in CD8	0.3989	0.2252

Figure 2a

Statistics: Mann-Whitney test		mean ± standard deviations	
	p value (two-tailed)	< 50	≥ 50
<*S.au* Blood>			
%IFNγ in CD4	0.0290	5.200 ± 3.428	1.474 ± 1.108
%IFNγ in CD8	>0.9999	17.82 ± 21.18	16.89 ± 17.19
<*S.au* Skin>			
%IFNγ in CD4	0.8513	22.80 ± 26.11	14.17 ± 9.922
%IFNγ in CD8	0.9451	29.58 ± 22.76	29.97 ± 22.39
<*C.alb* Blood>			
%IFNγ in CD4	0.0020	6.580 ± 3.493	1.526 ± 0.996
%IFNγ in CD8	>0.9999	24.38 ± 13.65	26.52 ± 19.98
<*C.au* Skin>			
%IFNγ in CD4	0.9143	8.480 ± 7.620	10.17 ± 10.02
%IFNγ in CD8	0.3524	31.15 ± 26.35	36.19 ± 20.43
<*S.au* Blood>			
%IL-17A in CD4	0.0140	1.806 ± 1.271	0.540 ± 0.415
%IL-17A in CD8	0.7238	0.806 ± 1.140	0.864 ± 0.925
<*S.au* Skin>			
%IL-17A in CD4	0.1703	1.683 ± 0.571	4.398 ± 3.381
%IL-17A in CD8	0.5769	0.926 ± 0.660	0.986 ± 0.710
<*C.alb* Blood>			
%IL-17A in CD4	0.0310	4.662 ± 2.514	1.672 ± 1.080
%IL-17A in CD8	0.0290	4.064 ± 1.994	1.586 ± 1.048
<*C.alb* Skin>			
%IL-17A in CD4	0.7619	4.070 ± 2.099	6.553 ± 6.469
%IL-17A in CD8	0.6571	3.243 ± 3.741	1.803 ± 1.305

Figure 3b, 3c, 3d

Statistics: Spearman rank correlation coefficient

	p value (two-tailed)	*r* value
Figure 3b blood	0.2838	-0.2842
Figure 3b skin	0.8974	0.03535
	p value (two-tailed)	*r* value
Figure 3c blood	0.0130	-0.6141
Figure 3c skin	0.8887	0.03829
	p value (two-tailed)	*r* value
Figure 3d blood	0.0652	0.4742
Figure 3d skin	0.5386	-0.1654

Figure 3e

Statistics: 2-way ANOVA followed by Tukey multiple comparisons test

	p value		
	Young vs Middle	Young vs Elderly	Middle vs Elderly
<Blood>			
Top 1	0.7807	0.9067	0.9690
Top 5	0.0959	0.3359	0.7884
Top 10	0.0299	0.1146	0.8418
Top 15	0.0133	0.0520	0.8660
Top 20	0.0073	0.0320	0.8604
<Skin>			
Top 1	0.9997	0.8612	0.8610
Top 5	0.3163	0.9913	0.4125
Top 10	0.1252	0.7542	0.452
Top 15	0.1085	0.7525	0.4151
Top 20	0.1136	0.7632	0.4173

Figure 4b

Statistics: Spearman rank correlation coefficient

	p value (two-tailed)	*r* value
Blood	0.0065	0.6613
Skin	0.1216	0.4035

Supplementary Figure 1a

Statistics: Spearman rank correlation coefficient

	p value (two-tailed)	*r* value
CD3 in blood	0.5958	-0.2132

Supplementary Figure 1b

Statistics: Spearman rank correlation coefficient

	p value (two-tailed)	*r* value
CD4/CD8 in blood (Swedish)	0.6676	0.1117
CD4/CD8 in blood (Japanese)	0.7458	0.08461
CD4/CD8 in epidermis (Japanese)	0.0348	-0.6484
CD4/CD8 in dermis (Japanese)	0.4284	-0.2648

Supplementary Figure 1c

Statistics: Spearman rank correlation coefficient

	p value (two-tailed)	*r* value
<%$CD69^+CD103^+$ in CD4>		
Epidermis	0.9410	-0.0177
Dermis	0.7001	0.09187
<%$CD49a^+$ in $CD69^+CD103^+CD4$>		
Epidermis	0.4712	0.1709
Dermis	0.8180	0.05497

Supplementary Figure 1d

Statistics: Spearman rank correlation coefficient

	p value (two-tailed)	*r* value
<Whole skin>		
%$CD69^+CD103^+$ in CD4	0.8142	0.0546
%$CD69^+CD103^+$ in CD8	0.0224	0.4953
<%$CD49a^+$ in $CD69^+CD103^+CD4$>		
Epidermis	0.4532	0.2511
Dermis	0.7075	-0.1279
<%$CD49a^+$ in $CD69^+CD103^+CD8$>		
Epidermis	0.0266	0.6728
Dermis	0.0875	0.5434

Supplementary Figure 1e

Statistics: Spearman rank correlation coefficient

	p value (two-tailed)	*r* value
<Blood>		
%Naïve T in CD4	0.0234	-0.7212
%T_{CM} in CD4	0.2768	0.3818
%T_{MM} in CD4	0.0734	0.6000
%T_{EM} in CD4	0.0347	0.6848
%Naïve T in CD8	0.0088	-0.7939
%T_{CM} in CD8	0.3129	0.3576
%T_{MM} in CD8	0.2182	0.4303
%T_{EM} in CD8	0.0149	0.7576
<Whole skin with IL-2/IL-15>		
%T_{CM} in CD4	0.8241	-0.05882
%T_{MM} in CD4	0.8315	0.05637
%T_{EM} in CD4	0.4727	0.1863
%T_{CM} in CD8	0.0244	-0.5490
%T_{MM} in CD8	0.8020	-0.0662
%T_{EM} in CD8	0.0442	0.4975

Supplementary Figure 1f

Statistics: Mann-Whitney test

	p value (two-tailed)	*r* value
%IFNγ in CD4	0.8881	0.07699
%IL-17A in CD4	0.3277	0.2402
%TNF in CD4	0.9546	-0.3077
%IFNγ in CD8	0.4559	0.2773
%IL-17A in CD8	0.4338	-0.3376
%TNF in CD8	0.4559	0.1914

Supplementary Figure 1g

Statistics: Spearman rank correlation coefficient

	p value (two-tailed)	*r* value
%IL-13 in CD4	0.8845	-0.05488
%IL-13 in CD8	0.4842	-0.2492

Supplementary Figure 1h

Statistics: Mann-Whitney test		mean ± standard deviations	
	p value (two-tailed)	< 50	≥ 50
<*S.au* Blood>			
%CFSE low in CD4	0.3636	2.410 ± 4.065	4.069 ± 3.967
%CFSE low in CD8	0.9241	2.024 ± 3.024	1.371 ± 1.308
<*C.alb* Blood>			
%CFSE low in CD4	0.6324	4.442 ± 6.746	6.940 ± 7.420
%CFSE low in CD8	0.7902	1.408 ± 1.769	2.651 ± 2.794
<*S.au* Skin>			
%Ki-67 in CD4	0.8740	5.493 ± 4.578	5.676 ± 3.661
%Ki-67 in CD8	0.7333	7.373 ± 5.175	5.521 ± 3.735
<*C.alb* Skin>			
%Ki-67 in CD4	0.9143	8.480 ± 7.620	10.17 ± 10.02
%Ki-67 in CD8	0.7619	4.188 ± 3.141	7.855 ± 7.507

Supplementary Figure 2a

Statistics: Mann-Whitney test		mean ± standard deviations	
	p value (two-tailed)	< 50	≥ 50
%CD69^{+}103^{+} in CD4	0.2991	34.63 ± 8.362	29.54 ± 10.81
%CD69^{+}103^{+} in CD8	0.6260	43.75 ± 12.61	39.14 ± 10.32
%IFNγ in CD4	0.4140	21.45 ± 7.900	25.29 ± 10.25
%IFNγ in CD8	0.4140	84.03 ± 9.987	78.99 ± 10.44
%IL-17A in CD4	0.1749	8.069 ± 6.918	78.99 ± 10.44
%IL-17A in CD8	0.2359	4.096 ± 4.846	1.409 ± 1.314

Supplementary Figure 2b

Statistics: Mann-Whitney test		mean ± standard deviations	
	p value (two-tailed)	< 50	≥ 50
%CD69^{+}103^{+} in CD4	0.7000	29.86 ± 8.682	24.99 ± 12.49
%CD69^{+}103^{+} in CD8	0.7000	36.68 ± 12.33	32.05 ± 6.287
%IFNγ in CD4	0.4000	20.37 ± 10.01	27.85 ± 10.34
%IFNγ in CD8	0.4000	77.69 ± 3.001	84.40 ± 10.64
%IL-17A in CD4	>0.9999	2.273 ± 1.152	4.395 ± 3.600
%IL-17A in CD8	0.6286	1.030 ± 0.946	2.008 ± 1.510
