# Supplementary material for: Skin T cells maintain their diversity and functionality in the elderly
Source: Commun Biol. 2021 Jan 4;4:13. doi: 10.1038/s42003-020-01551-7 (PMC7782613; doi:10.1038/s42003-020-01551-7)
Supplement: Supplementary file 3 — Description of Additional Supplementary Files [file 42003_2020_1551_MOESM3_ESM.pdf]

Description of Additional Supplementary Files

File Name: Supplementary Data 1
Description: Raw data for the graphs in Figure 1

File Name: Supplementary Data 2
Description: Raw data for the graphs in Figure 2

File Name: Supplementary Data 3
Description: Raw data for the graphs in Figure 3

File Name: Supplementary Data 4
Description: Raw data for the graphs in Figure 4

File Name: Supplementary Data 5
Description: Raw data for the graphs in Supplementary Figure 1

File Name: Supplementary Data 6
Description: Raw data for the graphs in Supplementary Figure 2
