# Supplementary material for: Skin T cells maintain their diversity and functionality in the elderly
Source: Commun Biol. 2021 Jan 4;4:13. doi: 10.1038/s42003-020-01551-7 (PMC7782613; doi:10.1038/s42003-020-01551-7)
Supplement: Supplementary file 4 — Supplementary Data 1 [file 42003_2020_1551_MOESM4_ESM.pdf]

a

CD3

Age	Epidermis	Dermis	Whole skin
57	3.0	5.0	8.0
20	3.0	8.0	11.0
72	4.0	2.0	6.0
68	7.0	8.0	15.0
40	2.0	5.0	7.0
80	7.0	3.0	10.0
85	9.0	5.0	14.0
36	3.0	4.0	7.0
92	9.0	13.0	22.0

CD4

Age	Epidermis	Dermis	Whole skin
57	1.0	1.7	2.7
20	1.0	5.0	6.0
72	2.0	1.0	3.0
68	3.0	6.0	9.0
40	1.0	3.0	4.0
80	4.0	2.3	6.3
85	5.0	1.0	6.0
36	1.0	2.0	3.0
92	4.0	9.0	13.0

CD8

Age	Epidermis	Dermis	Whole skin
57	2.0	3.0	5.0
20	2.0	3.0	5.0
72	2.0	1.3	3.3
68	4.0	2.0	6.0
40	1.0	1.7	2.7
80	3.0	1.0	4.0
85	4.0	4.0	8.0
36	2.0	2.0	4.0
92	5.0	4.0	9.0

b

Age	Epidermis	Dermis
49	3.8186813	2.705556
49	2.6095238	2.004651
70	1.4713897	0.895954
69	1.5314465	0.532847
41	1.8571429	2.608295
45	3.0772947	1.017647
40	2.0868167	1.613260
57	1.3300971	1.342960
51	3.1269036	1.610837
44	1.7977099	2.554455
40	2.2384342	0.970639
45	0.5093697	0.604770
76	0.3487545	0.124557
75	0.6918239	0.844633
23	5.4677419	2.218884
31	1.3029491	0.989218
61	0.4672414	15.82353
28	2.7329546	3.380000
34	3.5816327	2.243655
28	1.4323529	0.437819

c

Skin CD8

Age	Epidermis	Dermis
49	62.5	20.1
49	44.2	27.6
70	3.35	54.6
69	19.1	24.2
41	57.2	12.2
45	74.3	19.6
40	39.9	11.4
57	59.7	16.1
51	65.9	18.5
44	81.5	18.5
40	49.9	18.1
45	21.5	2.2
76	35.7	15.5
75	72.9	7.99
23	43.2	6.54
31	57.3	6.85
61	55.2	15.6
28	31.5	13.0
34	54.2	14.1
28	52.6	12.8

Skin CD8

Age	Epidermis	Dermis
49	68.6	2.41
49	69.6	1.32
70	63.1	2.24
69	55.2	5.12
41	78.1	2.56
45	60.0	1.12
40	54.8	1.03
57	79.9	5.27
51	45.3	0.48
44	78.8	22.0
40	70.4	45.0
45	83.3	38.3
76	91.7	4.55
75	74.6	23.7
23	53.4	11.9
31	68.5	25.0
61	76.4	39.1
28	9.59	0.87
34	33.5	9.28
28	47.7	11.0

d

Epidermis

	CD4 IFNγ		CD8 IFNγ		CD4 IL-17A		CD8 IL-17A		CD4 TNF		CD8 TNF	
Age	Epidermis	Dermis	Epidermis	Dermis	Epidermis	Dermis	Epidermis	Dermis	Epidermis	Dermis	Epidermis	Dermis
49	5.61	9.44	41.1	59.1	5.14	3.25	0.00	0.17	41.1	57.3	57.1	79.0
49	8.82	10.5	51.9	44.7	7.35	0.00	3.85	3.95	69.9	43.4	75.0	73.7
70	14.1	8.32	30.6	7.48	1.41	0.71	0.35	0.21	28.3	21.2	25.3	9.43
69	7.62	19.0	21.9	17.7	2.23	1.47	0.85	0.17	19.3	41.6	59.5	61.7
41	24.7	4.92	26.6	9.98	11.0	2.64	1.85	0.53	37.8	19.5	40.2	14.5
45	4.26	8.21	23.4	38.9	2.78	0.99	1.14	0.27	43.7	37.9	44.0	96.1
40	14.9	10.4	20.2	29.3	0.79	0.74	4.92	1.03	43.5	31	39.3	54.6
57	4.66	5.20	10.1	45.7	3.21	0.75	1.16	0.00	25.7	26.3	38.4	93.3
51	20.0	5.47	38.9	26.0	10.5	2.14	3.79	0.64	60.7	28.6	73.0	96.1
44	0.84	4.33	13.4	30.7	7.14	2.02	3.03	0.89				
40	0.13	2.79	1.79	9.94	1.13	6.75	0.15	0.68				
75	1.83	2.21	2.00	11.0	10.0	2.00	0.00	0.80	9.0	15.0	9.0	28.0
23	12.2	16.4	41.4	40.1	13.5	7.15	2.96	2.40	62.5	67.0	82.8	86.6
31	3.69	23.4	52.0	56.1	32.5	5.44	8.19	0.36	85.5	73.3	89.0	87.7
61	23.1	18.6	80.0	42.8	19.7	6.21	2.22	0.00	72.0	70.0	94.0	97.1
28	0.48	11.4	9.48	24.0	8.56	3.74	3.88	1.31	33.3	43.2	47.8	77.1
34	0.071	0.81	3.32	3.57	1.00	2.00	0.20	0.80	5.0	10.0	10.0	17.0
28	6.30	4.52	21.5	16.1	4.57	5.59	3.70	1.17	52.8	58.3	60.0	76.0

Whole skin with IL-2/IL-15

Age	CD4 IFNγ	CD8 IFNγ	Age	CD4 IL-17A	CD8 IL-17A	Age	CD4 TNF	CD8 TNF
72	38.4	70.3	72	7.32	3.96	72	91.4	91.5
74	26.5	87.7	87	5.68	0.66	84	76.5	87.9
64	37.8	59.3	74	21.7	4.50	87	77.0	79.0
96	14.1	69.8	64	16.8	0.15	74	90.0	95.6
30	34.3	32.3	96	1.88	2.50	64	81.6	87.5
87	17.3	81.0	30	6.20	4.90	96	91.0	95.0
86	15.4	93.0	87	3.81	1.99	30	80.0	89.0
83	29.8	85.6	86	4.13	0.44	87	87.0	96.5
42	31.9	25.8	83	12.0	1.29	86	61.5	60.9
26	15.4	24.8	42	9.73	23.6	83	68.9	81.5
17	25.0	60.2	51	13.0	15.0	42	68.6	59.8
48	14.4	71.1	26	19.0	10.7	51	61.0	78.4
88	35.0	53.0	17	11.1	6.00	26	46.3	71.2
70	18.0	52.2	85	2.40	6.40	17	76.5	88.3
83	20.0	64.0	48	15.6	8.47	85	86.6	89.0
63	19.0	60.0	63	1.37	1.86	48	77.7	95.4
65	31.0	48.0	65	3.57	0.00			
90	11.1	68.0	90	1.88	1.23			
			88	1.25	0.83			
			70	7.70	0.80			
			83	1.31	2.44			
