# Supplementary material for: Skin T cells maintain their diversity and functionality in the elderly
Source: Commun Biol. 2021 Jan 4;4:13. doi: 10.1038/s42003-020-01551-7 (PMC7782613; doi:10.1038/s42003-020-01551-7)
Supplement: Supplementary file 5 — Supplementary Data 2 [file 42003_2020_1551_MOESM5_ESM.pdf]

a

*S.au*

IFNγ

<50

| | | | | | |
|---|---|---|---|---|---|
| Blood CD4 | 10.32 | 5.11 | 6.27 | 2.87 | 1.43 |
| Blood CD8 | 16.77 | 9.10 | 7.84 | 54.37 | 1.03 |
| Skin CD4 | 8.18 | 59.0 | 24.0 | 0.00 | |
| Skin CD8 | 11.28 | 39.76 | 56.9 | 10.37 | |

≥ 50

| | | | | | | | | | | |
|---|---|---|---|---|---|---|---|---|---|---|
| Blood CD4 | 1.69 | 2.15 | 2.10 | 2.21 | 2.84 | 0.11 | 0.00 | 0.04 | | |
| Blood CD8 | 19.89 | 54.2 | 30.47 | 15.0 | 13.85 | 1.18 | 0.89 | 0.68 | | |
| Skin CD4 | 37.36 | 8.64 | 22.35 | 17.50 | 10.20 | 12.90 | 3.53 | 7.09 | 18.40 | 2.43 |
| Skin CD8 | 11.10 | 33.57 | 25.00 | 6.13 | 67.74 | 59.12 | 14.58 | 60.00 | 20.29 | 25.00 |

*C. alb*

IFNγ

<50

| | | | | | |
|---|---|---|---|---|---|
| Blood CD4 | 8.27 | 4.26 | 11.58 | 6.13 | 2.66 |
| Blood CD8 | 13.50 | 14.35 | 17.07 | 44.60 | 32.40 |
| Skin CD4 | 2.09 | 3.70 | 18.97 | 9.16 | |
| Skin CD8 | 15.53 | 21.43 | 70.5 | 17.14 | |

≥ 50

| | | | | | | | | | |
|---|---|---|---|---|---|---|---|---|---|
| Blood CD4 | 1.99 | 2.33 | 3.24 | 2.4 | 1.27 | 0.49 | 0.72 | 0.52 | 0.77 |
| Blood CD8 | 34.80 | 16.00 | 58.30 | 43.70 | 41.40 | 30.60 | 3.80 | 4.10 | 6.00 |
| Skin CD4 | 28.00 | 5.26 | 12.94 | 3.33 | 11.49 | 0.00 | | | |
| Skin CD8 | 22.00 | 22.86 | 70.80 | 38.24 | 46.86 | 16.4 | | | |

*S.au*

IL-17A

<50

| | | | | | |
|---|---|---|---|---|---|
| Blood CD4 | 3.87 | 0.88 | 2.15 | 0.81 | 1.32 |
| Blood CD8 | 0.42 | 0.22 | 0.34 | 2.84 | 0.21 |
| Skin CD4 | 1.92 | 1.93 | 2.05 | 0.83 | |
| Skin CD8 | 1.50 | 0.22 | 0.51 | 1.48 | |

≥ 50

| | | | | | | | | | | | |
|---|---|---|---|---|---|---|---|---|---|---|---|
| Blood CD4 | 0.27 | 1.04 | 0.81 | 1.36 | 0.15 | 0.41 | 0.14 | 0.17 | 0.44 | | |
| Blood CD8 | 1.88 | 0.61 | 0.81 | 2.86 | 0.21 | 0.54 | 0.00 | 0.19 | 0.68 | | |
| Skin CD4 | 1.83 | 2.30 | 3.29 | 1.02 | 2.50 | 0.45 | 6.45 | 10.26 | 2.36 | 12.09 | 5.83 |
| Skin CD8 | 1.40 | 0.00 | 0.70 | 0.00 | 0.97 | 1.61 | 1.46 | 0.00 | 1.67 | 1.19 | 1.85 |

*C.alb*

IL-17A

<50

| | | | | | |
|---|---|---|---|---|---|
| Blood CD4 | 7.69 | 4.26 | 0.77 | 5.26 | 5.33 |
| Blood CD8 | 6 | 1.82 | 6.29 | 3.51 | 2.7 |
| Skin CD4 | 4.63 | 6.16 | 4.33 | 1.16 | |
| Skin CD8 | 0 | 2.88 | 8.57 | 1.52 | |

≥ 50

| | | | | | | | | | |
|---|---|---|---|---|---|---|---|---|---|
| Blood CD4 | 1.27 | 1.55 | 2.52 | 3.09 | 0.00 | 2.17 | 0.72 | 2.96 | 0.77 |
| Blood CD8 | 2.96 | 2.44 | 2.78 | 2.19 | 0.68 | 1.12 | 0.00 | 1.40 | 0.70 |
| Skin CD4 | 10.00 | 17.03 | 2.80 | 8.21 | 1.28 | 0.00 | | | |
| Skin CD8 | 0.00 | 2.86 | 1.77 | 3.68 | 1.14 | 1.37 | | | |
