# Supplementary material for: Skin T cells maintain their diversity and functionality in the elderly
Source: Commun Biol. 2021 Jan 4;4:13. doi: 10.1038/s42003-020-01551-7 (PMC7782613; doi:10.1038/s42003-020-01551-7)
Supplement: Supplementary file 6 — Supplementary Data 3 [file 42003_2020_1551_MOESM6_ESM.pdf]

	b		c		d	
Age	Blood	Skin	Blood	Skin	Blood	Skin
46	21859	4018	0.912602	0.519294	79	74
57	12186	9625	0.845432	0.835565	59	79
73	11667	5106	0.734162	0.656483	49	71
57	24575	860	0.827018	0.626599	71	81
66	9872	872	0.603006	0.476276	37	76
41	22040	2011	0.920657	0.568771	73	81
79	10503	3729	0.775764	0.712777	61	60
20	11407	546	0.777909	0.752529	67	83
48	23834	3216	0.852616	0.336519	80	76
90	14324	2789	0.668574	0.704038	81	83
83	11954	896	0.861136	0.786386	70	67
90	10844	1106	0.815360	0.568491	66	70
88	15136	7629	0.680319	0.484414	80	84
42	23704	6523	0.855587	0.380836	80	75
84	16459	3367	0.743318	0.265311	76	69
26	13674	3933	0.895355	0.500421	74	74

e

% top clones occupy

Blood	Young						Middle					Elderly				
top 1	2.04	0.76	2.03	0.49	1.39	1.01	3.37	4.76	1.45	8.93	6.43	7.54	1.34	2.03	5.69	1.46
top 5	5.23	1.87	4.67	1.52	3.3	2.24	7.92	12.20	5.76	32.57	15.35	24.04	3.71	5.35	15.7	6.06
top 10	6.39	2.45	7.10	2.58	4.50	2.96	9.79	15.01	9.02	40.80	18.90	32.60	5.29	7.17	22.63	9.72
top 15	6.80	2.92	9.04	3.44	5.39	3.49	10.77	17.09	11.59	45.95	20.85	37.70	6.48	8.70	26.56	12.09
top 20	7.23	3.36	10.78	4.16	6.08	3.98	11.49	19.00	13.89	49.03	22.43	40.35	7.48	10.04	28.84	14.08

Skin	Young						Middle					Elderly				
top 1	14.60	22.94	13.85	23.03	13.75	7.19	1.36	6.10	13.35	52.66	4.50	7.52	15.72	34.01	7.15	48.17
top 5	42.60	47.78	29.57	78.89	57.11	27.70	5.00	19.29	39.66	62.24	17.42	25.85	24.67	48.82	39.80	89.23
top 10	50.98	60.51	39.43	91.03	84.25	43.71	7.45	25.75	53.57	67.21	28.54	35.53	31.02	60.00	45.41	90.76
top 15	56.60	67.43	47.25	91.59	88.6	54.98	9.25	31.10	61.90	70.78	35.51	40.40	36.19	65.46	59.46	91.48
top 20	61.46	70.55	53.24	91.90	89.63	63.46	10.87	35.86	68.67	73.38	40.99	43.62	40.15	68.77	69.51	91.92
