# Supplementary material for: Skin T cells maintain their diversity and functionality in the elderly
Source: Commun Biol. 2021 Jan 4;4:13. doi: 10.1038/s42003-020-01551-7 (PMC7782613; doi:10.1038/s42003-020-01551-7)
Supplement: Supplementary file 7 — Supplementary Data 4 [file 42003_2020_1551_MOESM7_ESM.pdf]

b		Blood	Skin
Age		% overlap/unique	
	46	0.03203	0.17422
	57	0.06565	0.08312
	73	0.54856	1.25343
	57	0.17498	5.00000
	66	0.21273	2.40826
	41	0.32668	3.58031
	79	0.66648	1.87718
	20	0.14027	2.93041
	48	0.08811	0.65299
	90	1.15192	5.91610
	83	0.31789	4.24108
	90	0.31354	3.07415
	88	1.28172	2.54293
	42	0.03797	0.13798
	84	0.26126	1.27711
	26	0.13164	0.45767
