# Supplementary material for: Skin T cells maintain their diversity and functionality in the elderly
Source: Commun Biol. 2021 Jan 4;4:13. doi: 10.1038/s42003-020-01551-7 (PMC7782613; doi:10.1038/s42003-020-01551-7)
Supplement: Supplementary file 8 — Supplementary Data 5 [file 42003_2020_1551_MOESM8_ESM.pdf]

a

Age	T cell number
23	1038.612
34	1767.161
40	2500.833
60	1929.471
63	1140.216
68	1592.649
81	1863.674
86	1300.471
89	870.2787
82	2153.802
104	1152.826
70	1356.641
48	1695.231
70	928.5905
35	1337.677
54	1344.193
73	1220.355

b

Swedish Blood

Age	CD4/CD8
49	5.0822785
49	1.8865672
70	1.1239316
69	4.4166667
41	3.9656863
45	1.1666667
40	2.1157556
57	1.596730
51	3.344660
45	2.1233333
76	1.9009009
75	0.0107595
31	1.3737864
61	1.5555556
28	1.8974359
34	0.0518562
28	0.0074777

Japanese Blood

Age	CD4/CD8
23	2.025
34	0.842
40	7.099
60	3.124
63	3.278
68	2.839
81	1.816
86	5.117
89	1.436
82	2.738
104	2.647
70	4.893
48	3.638
70	2.327
35	0.781
54	1.756
73	0.801

	Epidermis	Dermis
Age	CD4/CD8	CD4/CD8
47	4.13	1.18
77	3.20	2.72
31	5.11	1.72
70	3.10	5.99
17	5.33	9.55
48	2.00	0.20
85	4.20	2.13
48	7.38	1.84
80	0.64	0.68
77	2.00	1.50
89	0.88	1.15

c

	%CD69+CD103+		%CD49a+inCD4+CD69+CD103+	
Age	Epidermis	Dermis	Epidermis	Dermis
49	39.3	12.6	9.52	5.94
49	44.9	12.5	1.64	0.00
70	63.4	6.83	16.1	8.13
69	6.32	24.5	14.7	20.0
41	35.7	9.31	6.15	8.07
45	64.3	15.6	15.9	8.09
40	46.6	8.12	36	22.4
57	25.9	6.47	16.9	5.74
51	71.5	17.8	9.34	4.51
44	53.7	20.0	46.4	19.2
40	25.5	11.1	2.86	6.72
45	34.3	18.5	31.1	15.4
76	37.9	12.3	12.1	5.71
75	61.5	15.4	78.3	21.3
23	42.9	9.82	0.51	0.47
31	67.0	14.7	68.5	24.7
61	46.9	25.5	13	11.8
28	37.9	16.3	9.95	7.67
34	53.0	13.1	1.55	1.17
28	52.6	12.8	47.7	11.0

d

%CD69+CD103+

Age	in CD4	in CD8
72	49.29	46.72
56	32.48	31.37
87	32.25	50.00
74	30.38	68.41
64	26.01	52.83
96	49.45	36.00
83	22.39	40.00
81	36.60	35.00
51	38.85	45.45
70	27.01	46.54
63	28.00	49.77
64	39.29	25.28
90	22.20	35.00
88	36.91	34.06
70	26.05	25.00
83	12.00	37.08
30	30.95	17.86
42	21.82	6.21
26	51.58	18.42
17	24.28	20.00
48	21.93	17.27

	%CD49a+inCD4+CD69+CD103+		%CD49a+inCD8+CD69+CD103+	
Age	Epidermis	Dermis	Epidermis	Dermis
47	7.63	3.51	71.74	61.54
77	15.09	11.03	51.16	67.57
31	12.18	15.27	64.52	50.00
70	27.63	26.87	84.69	62.22
17	11.41	68.33	46.43	11.45
48	42.57	60.38	44.05	67.44
85	6.48	11.21	72.27	79.46
48	10.23	20.59	51.16	58.11
80	43.90	30.00	90.94	30.00
77	5.35	1.86	71.19	35.71
89	56.23	38.92	94.38	72.88

e

Blood

	CD4				CD8			
Age	Naive	T_{CM}	T_{MM}	T_{EM}	Naive	T_{CM}	T_{MM}	T_{EM}
27	53.78	7.33	2.79	19.92	78.41	0.45	5.52	0.00
34	69.84	1.74	4.83	19.98	79.65	0.00	9.23	0.19
45	65.78	10.94	0.90	8.40	73.13	0.14	6.42	0.84
56	57.23	8.06	2.17	23.15	68.46	0.85	12.5	2.75
70	37.52	13.48	1.45	22.11	49.56	2.53	18.93	2.53
72	10.33	41.22	6.72	37.75	27.71	3.69	25.42	1.47
73	63.93	8.23	3.58	6.98	74.41	1.09	7.03	1.41
80	28.45	16.05	4.39	40.66	37.35	3.60	16.45	2.29
85	15.71	18.19	5.34	38.27	39.35	2.36	22.17	2.31
89	7.76	2.16	11.51	66.90	12.62	0.06	54.17	1.12

Skin

	CD4			CD8		
Age	T_{CM}	T_{MM}	T_{EM}	T_{CM}	T_{MM}	T_{EM}
68	7.67	5.26	45.2	9.49	15.14	62.15
33	9.37	10.97	31.51	7.63	19.24	56.95
72	4.08	4.39	27.8	4.87	9.72	61.69
56	6.88	11.15	67.87	7.82	3.72	36.92
87	7.59	9.79	49.75	1.64	7.98	84.25
74	4.18	10.71	43.75	0.24	7.69	88.46
64	0.64	1.61	93.88	1.42	5.84	86.64
96	3.06	2.34	25.27	2.14	11.88	81.68
30	12.5	23.96	31.35	14.58	8.09	51.47
83	18.75	6.78	33.99	9.68	1.92	57.73
42	2.46	2.15	25.56	10.55	14.97	58.41
81	3.77	15.54	75.64	2.99	7.89	83.92
47	3.37	1.22	24.04	3.14	2.04	62.24
51	2.34	1.65	65.08	3.33	4.65	70.66
26	20.45	7.25	31.41	11.54	9.89	60.55
73	1.29	0.93	53.79	5.57	4.25	68.71
70	2.68	11.35	42.7	3.57	16.18	69.85

f

Age	CD4 IFNγ	CD8 IFNγ	CD4 IL-17A	CD8 IL-17A	CD4 TNF	CD8 TNF
49	10.8	40.1	0.78	0.31	57.0	42.0
49	10.5	54.5	0.75	0.055	47.5	52.5
70	32.3	86.0	1.83	0.059	74.7	69.4
69	0.95	2.39	0.17	0.00	9.01	4.02
41	3.49	7.21	0.45	0.00	26.8	17.5
45	15.3	46.3	0.51	0.027	77.7	71.5
40	7.58	15.7	0.12	0.00	61.4	22.9
57	12.1	49.9	0.27	0.0073	83.7	72.7
51	5.87	34.3	0.46	0.11	75.1	61.9
75	13.7	32.0	0.00	0.00	18.0	35.0
31	13.9	59.1	0.41	1.26	84.9	73.2
61	13.9	56.4	0.73	0.032	82.0	72.9
28	2.45	16.2	1.11	0.070	65.6	18.3
34	9.59	20.2	1.00	0.012	19.0	37.0
28	43.7	25.7	1.54	0.066	88.9	34.0

g

	IL-13	
Age	CD4	CD8
74	3.92	2.60
64	3.00	3.80
96	1.50	0.58
30	1.50	3.10
83	7.00	6.40
42	1.70	1.20
26	3.00	2.60
76	0.20	0.86
63	5.14	2.79
66	0.90	1.35

h

CFSE low

S.au									
<50									
CD4	1.03	9.64	0.99	0.39	0.00				
CD8	1.49	7.68	0.28	0.49	0.18				
≥ 50									
CD4	7.10	4.43	12.91	3.95	0.91	0.11	3.28	3.25	0.68
CD8	2.86	3.16	2.88	1.07	0.00	0.00	1.49	0.88	0.00
C. alb									
<50									
CD4	3.86	16.22	1.54	0.59	0.00				
CD8	1.94	4.28	0.32	0.50	0.00				
≥ 50									
CD4	15.10	18.93	14.97	1.99	0.22	0.00	0.43	4.81	6.01
CD8	6.75	4.84	2.51	0.00	0.00	0.00	0.00	3.70	6.06

%Ki-67

S.au											
<50											
CD4	3.46	8.90	9.61	0.00							
CD8	5.26	10.73	12.4	1.10							
≥ 50											
CD4	6.32	5.46	8.64	8.92	7.47	2.50	6.73	0.00	1.28	12.1	3.02
CD8	3.50	11.1	6.99	8.33	5.18	1.64	12.41	1.85	2.08	3.30	4.35
C. alb											
<50											
CD4	9.26	6.16	2.87	0.23							
CD8	7.14	2.88	6.35	0.38							
≥ 50											
CD4	18.0	3.10	4.55	1.03	8.23	2.06					
CD8	0.00	5.71	0.88	15.4	18.29	6.85					
