# Supplementary material for: Skin T cells maintain their diversity and functionality in the elderly
Source: Commun Biol. 2021 Jan 4;4:13. doi: 10.1038/s42003-020-01551-7 (PMC7782613; doi:10.1038/s42003-020-01551-7)
Supplement: Supplementary file 9 — Supplementary Data 6 [file 42003_2020_1551_MOESM9_ESM.pdf]

a

Head	CD4	CD8
Age	CD69+103+	CD69+103+
56	32.48	31.37
87	32.25	50.00
74	30.38	68.41
96	49.45	36.00
83	22.39	40.00
81	36.60	35.00
51	38.85	45.45

Head	CD4	CD8
Age	IFNγ	IFNγ
74	26.54	87.70
96	14.07	69.79
86	15.37	93.00
83	29.81	85.61

Head	CD4	CD8
Age	IL-17A	IL-17A
87	5.68	0.66
74	21.7	4.5
96	1.88	2.5
87	3.81	1.99
86	4.13	0.44
83	11.95	1.29
51	13	15
85	2.4	6.4

Trunk	CD4	CD8
Age	CD69+103+	CD69+103+
72	49.29	46.72
64	26.01	52.83
70	27.01	46.54
63	28.00	49.77
65	39.29	25.28
90	22.20	35.00
88	36.91	34.06
70	26.05	25.00
83	12.00	37.08

Trunk	CD4	CD8
Age	IFNγ	IFNγ
63	19.00	75.86
65	31.00	76.05
90	11.12	81.15
88	35.00	92.50
70	18.00	82.01
83	20.00	92.77
72	38.38	70.33
64	37.82	59.29
87	17.27	80.96

Trunk	CD4	CD8
Age	IL-17A	IL-17A
63	1.37	1.86
65	3.57	0.00
90	1.88	1.23
88	1.25	0.83
70	7.70	0.80
83	1.31	2.44
72	7.32	3.96
64	16.8	0.15

b

	CD4	CD8
	CD69+103+	CD69+103+
Age	Flap	
63	28.00	49.77
65	39.29	25.28
90	22.20	35.00
Age	Transplant	
88	36.91	34.06
70	26.05	25.00
83	12.00	37.08

	CD4	CD8
	IFNγ	IFNγ
Age	Flap	
63	19.00	75.86
65	31.00	76.05
90	11.12	81.15
Age	Transplant	
88	35.00	92.50
70	18.00	82.01
83	20.00	92.77
72	38.38	70.33

	CD4	CD8
	IL-17A	IL-17A
Age	Flap	
63	1.37	1.86
65	3.57	0.00
90	1.88	1.23
Age	Transplant	
88	1.25	0.83
70	7.70	0.80
83	1.31	2.44
72	7.32	3.96
